# Estrogen Receptor 1 Gene Expression and Its Combination with Estrogen Receptor 2 or Aromatase Expression Predicts Survival in Non-Small Cell Lung Cancer

**DOI:** 10.1371/journal.pone.0109659

**Published:** 2014-10-13

**Authors:** Unai Aresti, Sergio Carrera, Eluska Iruarrizaga, Natalia Fuente, Ines Marrodan, Abigail Ruiz de Lobera, Alberto Muñoz, Aitziber Buque, Elizabeth Condori, Irene Ugalde, Begoña Calvo, Guillermo López Vivanco

**Affiliations:** 1 Oncology Research Laboratory, Cruces University Hospital/BioCruces Health Research Institute, Barakaldo, Bizkaia, Spain; 2 Medical Oncology Department, Cruces University Hospital, Barakaldo, Bizkaia, Spain; University Medical Center Hamburg-Eppendorf, Germany

## Abstract

The biological roles of estrogen receptor 1 (*ERS1*), estrogen receptor 2 (*ERS2*), and aromatase (*CYP19A1*) genes in the development of non-small cell lung cancer (NSCLC) is unclear, as is the use of their expression as a prognostic factor. The aim of this study was to investigate the prognostic value of estrogen receptors and aromatase mRNA expression, along with aromatase protein concentration, in resected NSCLC patients. Tumor and non-tumor lung tissue samples were analyzed for the mRNA expression of *ERS1*, *ERS2* and *CYP19A1* by RT-PCR. Aromatase concentration was measured with an ELISA. A total of 96 patients were included. *ERS1* expression was significantly higher in non-tumor tissue than in tumor samples. Two gene expression categories were created for each gene (and protein): high and low. *ERS1 high* category showed increased overall survival (OS) when compared to the low expression category. Aromatase protein concentration was significantly higher in tumor samples. Higher *ERS1* expression in tumor tissues was related to longer overall survival. The analysis of gene expression combinations provides evidence for longer OS when both *ERS1* and *ERS2* are highly expressed. *ESR1*, alone or in combination with *ERS2* or *CYP19A*1, is the most determining prognostic factor within the analyzed 3 genes. It seems that *ERS1* can play a role in NSCLC prognosis, alone or in combination with other genes such as *ERS2* or *Cyp19a1*. *ERS2* in combination with aromatase concentration could have a similar function.

## Introduction

Taking statistics from the USA as an example of the evolution of lung cancer in developed countries, it is clear that the prevention and cure of this particular type of cancer needs to improve. The projections for 2014 are 159,260 deaths due to lung cancer (27.2% of all cancer deaths) while this cancer causes more deaths than the sum of the three next most common cancers (colon, breast and prostate) [1]. Further, the evolution of the lung cancer five-year survival rates, estimated to be about 12%, 13% and 16% in the periods 1975–77, 1987–89 and 2001–2007 respectively [2], has been clearly unsatisfactory.

Estrogens are involved in the development of several types of cancer: breast, endometrial, ovarian, thyroid, and lung [3]–[8]. In the lung, estrogens induce cell proliferation through the activation of certain growth factor genes such as epidermal growth factor and insulin-like growth factor, mediators of mitogenesis in lung tumors [9]. They exert their function through two different specific estrogen receptors, estrogen receptor-α (ERα) and estrogen receptor-β (ERβ), members of the nuclear receptor superfamily of transcription factors [10], and through two different pathways, genomic and non-genomic, with the same biological effects: proliferation, growth, apoptosis, differentiation and angiogenesis. *ERS1* and *ERS2* genes are expressed in most of the more common cancers, lung, breast, digestive and endocrine, but their role can vary greatly depending on the tumor type [10]–[16].

Production of the two most abundant and important estrogens (estradiol and estrone) is catalyzed by cytochrome p450 19A1 (aromatase), the product of *CYP19A1* gene, that is active in lung tissues. In addition, there is a feedback loop, because estrogens induce the activation of epidermal growth factor that leads to an increase in aromatase expression and activity [17], in turn, enhancing estrogen production. Clearly, these facts indicate that aromatase plays a notable role in cancer development, and it has been hypothesized that its elevated expression indicates a poorer prognosis [18].

This work try to confirm (or reject) if any of these genes expressions is useful as prognostic tool and to clarify the disagreement between past studies based mainly in immunohistochemistry (IHC) methods [6], [12], [19], and more recent ones that have used real time PCR technique to determine gene expression [20], [21]. In this sense different expression levels groups will be established and related to survival.

## Materials and Methods

### 1.-Ethics Statement

This study was approved by the Ethics Committee for Clinical Research of Cruces University Hospital (CEIC/Hospital de Cruces) and was performed according to the declaration of Helsinki. Written informed consent was obtained from every patient after full explanation of the purpose and nature of all procedures used.

### 2.-Patients

Among 110 recruited patients, only 96 patients fulfilled the selection criteria. To be included patients should be diagnosed with NSCLC and underwent resection surgery; patients receiving neoadjuvant chemotherapy and patients whose primary tumor was not located in the lung were excluded. Their clinical characteristics are described in **Table 1**. For each participant, tumor and non-tumor fresh tissue samples were prepared by anatomopathologist. Samples were obtained through macro dissection and non-tumor tissue corresponded to adjacent normal lung tissues.

**Table 1:** Patient Characteristics.

		N = 96	%
**SEX**			
	Male/Female	81/15	84.4/15.6
**SMOKING STATUS**			
	Smoker	38	39.5%
	Formerly smoker	44	45.8%
	Non-smoker	7	7.3%
	Unknown	7	7.3%
**ADJUVANT CT** * **TREATMENT**			
	Yes/No	32/64	34.0%/66.0%
**RECURRENCE**			
	Yes/No	41/55	42.7%/57.3%
	Local/Metastatic (n = 41)	13/28	31.7%/68.3%
**HISTOLOGY**			
	Adenocarcinoma	41	42.7%
	Squamous	42	43.8%
	Bronchioalveolar	8	8.3%
	Others	5	5.2%
**PATHOLOGICAL STAGE**			
	I	41	42.7%
	II	32	33.3%
	III	21	21.9%
	IV	2	2.1%

*Chemotherapy.

### 3.-Techniques

#### 3.1-REAL TIME RT-PCR

Total RNA from tumor and non-tumor tissue was extracted with RNeasy Plus Mini Kit (Qiagen). One-step real-time RT-PCRs were performed using a Platinum Quantitative RT-PCR Thermoscript One-Step System and 7900HT Fast Real-Time PCR System (both from Life Technologies). Estrogen receptors and aromatase expression assays as well as RPLPO (endogenous control) were purchased from Life Technologies: *ERS1*/Hs00174860_m1, *ERS2*/Hs00230957_m1, *CYP19A1*/Hs00903411_m1 and RPLPO/NM_053275.3. Each assay amplifies a number of different transcripts. Detailed information about transcript length, associated protein length, probe exon-exon boundary, amplicon amplification and protein functionality is included in File S1.

Total RNA was added to obtain a concentration of 100 µg/µl in a final volume of 10 µl reaction. A standard time/temperature profile was used. A positive control was included in each plate: RNA samples extracted from MCF-7 cells (HTB22), purchased (november 2009) from ATCC. HTB22 was chosen as a reference (calibrator) sample for comparative quantization (by the Delta Delta Cq method). Gene expression is reported as RQ values obtained from comparative quantification, where RQ = Normalized Relative Quantity  = 2^−(ΔΔ Cq)^.

#### 3.2-ELISA

Enzyme-linked immunosorbent assay kits for human aromatase (ARO) (USCN Life Science Inc.) were used for aromatase protein detection. Protein was extracted from tumor and non-tumor samples, following this kit protocol. For this procedure, tissues were available from 85 patients. Protein concentration was measured in a Polar Star Microplate Reader (BMG Labtech) using the bicinchoninic acid protein method. Data were analyzed using *Four Parameter Logistic Fit*, developed by MyAssays, Analysis Software Solutions.

### 4-Statistical analysis

For statistical analysis, SPSS/v.17, Graphpad-Prism/v.3 and MedCalc/12.7.5 software packages were selected. In brief, these were used for calculating descriptive statistics, and for performing: Kolmogorov-Smirnov tests for normality, non-parametric tests (Mann-Whitney, Kruskal Wallis) when comparing medians, correlation, survival and Cox regression analysis. All tests were two sided and significance level was 0.05. For survival analysis, Kaplan-Meier curves were constructed and the significance was assessed using the Log Rank (Mantel-Cox) test. To assess the influence of variables on overall survival, Cox Regression analysis was performed. First, pairwise comparisons were made and any variables with a significance of less than 0.2 were retained in a second general analysis. Then, they were eliminated, one-by-one, if the significance exceeded the threshold of 0.05. The analysis was repeated after the removal of each variable. The overall survival (OS) was calculated from the date of surgery/resection to the date of last follow-up or death, in months.

## Results

### 1-*ERS1*

The first comparison, between non-tumor and tumor tissue *ERS1* levels obtained a p<0.0001, considered extremely significant. Statistics of RQ comparison results are shown in **Table 2**. As can be seen, *ERS1* gene expression was significantly lower in tumor than non-tumor tissues.

**Table 2:** Medians for the three genes (*ERS1*, *ERS2*, and *CYP19A1*) relative expression (RQ) measures, N = 96, and aromatase protein concentration, N = 85.

	*ERS1*		*ERS2*		*CYP19A1*		ARO*	
	T	NT	T	NT	T	NT	T	NT
Median	0.05	0.13	11.21	11.51	0.11	0.13	4.05	1.86
p-value		0.0001		0.3640		0.3205		0.0001

Also includes p-values for Man-Whitney tumor/non-tumor RQ comparison.

T = Tumor; NT = Non-tumor.

ARO* =  Protein concentration ELISA values, ng/ml.

**Figure 1:**
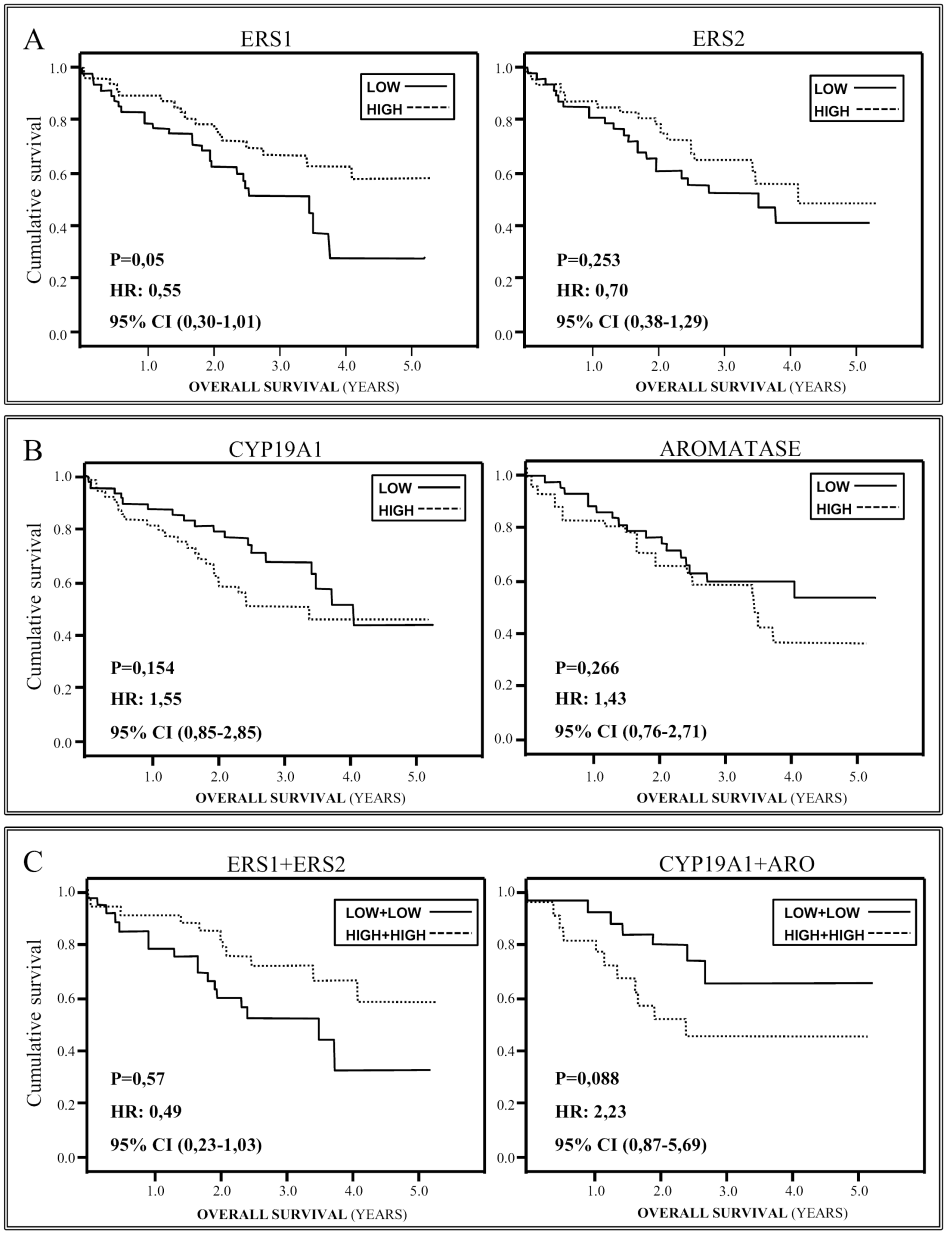
Kaplan-Meier survival curves when considering gene mRNA expression levels (*ERS1, ERS2* and *CYP19A1*), or protein concentration (ARO), as discriminating factor. The patient sample is divided into Low and High (lower and higher expression than median). Panels: a) *ERS1*and *ERS2*, b) *CYP19A1* and ARO, and c) *ERS1+ERS2* and CYP19A1 + ARO.

For analysis of the relationship between survival and *ERS1* gene expression, samples were split into two groups, according to the median expression levels (Low for lower values than median, high for higher values than median values Kaplan-Meier survival curves were calculated for both, revealing significant differences within the tumor samples; Log Rank = 0.050, **figure 1a**. For the tumor tissues, the median survival was 28.0 months in the lower *ERS1* expression group, and 34.0 months in the higher *ERS1* expression group (**Table 3**); a Mann-Whitney test showed this to be a significant difference, p = 0.042, pointing to a relationship between higher *ERS1*gene expression in tumor tissue and longer patient survival.

**Table 3:** Medians of overall survival (months) for the three genes, *ERS1*, *ERS2*, and *CYP19A1* (Low and High, N = 48) and aromatase protein, ARO (Low N = 43 and High N = 42), when categorizing their values into low and high expression values (lower and higher expression than median).

	*ERS1*		*ERS2*		*CYP19A1*		ARO	
	T	NT	T	NT	T	NT	T	NT
Lower	28.0	30.2	26.9	33.1	31.1	30.6	30.3	33.6
Higher	34.0	29.7	31.2	25.9	28.8	29.7	28.3	28.8
Log-rank	0.050	0.527	0.253	0.078	0.154	0.576	0.266	0.528
Man-Whitney	0.042	0.826	0.110	0.024	0.145	0.529	0.308	0.134

Kaplan-Meier Log Rank and Mann-Whitney p-values when comparing Low vs. High groups are also listed. T = Tumor; NT = Non-tumor.

To confirm, or rule out, on the basis of survival results whether gene expression was altered in the tumor samples, the same analysis was performed for non-tumor samples from the same patients, and no significant differences in survival were found between these subgroups; Log Rank = 0.524; p = 0.815.

*ERS1* expression in tumor samples was the only variable, within the three genes and the protein, which was significant when developing a Cox regression model: The total significance level was 0.031 for the model, p = 0.027 for the *ERS1* tumor mRNA level with an Exp (B) of 0.002. P-value for non-tumor tissue samples was 0.529, not significant, so it was not included in the model. As the ratio tumor/non-tumor, that passed first round, p = 0.105, but then appears not to be significant. Just histology (including merely adenocarcinomas and squamous-cell carcinomas) was also significant with a p = 0.007 and an Exp (B) of 0.462.

### 2-*ERS2*

*ERS2* gene expression was compared in the tumor/non-tumor tissues and in this case no significant differences were found (Mann-Whitney test, p = 0.364). It can be seen in **Table 2**, the gene expression results were very similar in the two types of tissue. Analyzing survival as a function of ERS2 gene expression level as previously (**Figure 1a**), no significant differences were found between Low and High expression subgroups of tumor (p = 0.253) or non-tumor (p = 0.078) samples. It was a notable trend toward better survival in patients with lower ERS2 levels in their non-tumor tissue samples, suggesting a possible lower expression/longer survival association.

Keeping these data in mind, the significance of differences in OS was also assessed with a Mann-Whitney test. As can be seen in **Table 3**, the pattern in tumor samples was the inverse of that in non-tumor samples: the median OS rates in low/tumor group were considerably lower than those in high/tumor group, though these differences were not significant (p = 0.11). In contrast, the median rates were considerably higher in the low/non-tumor group than the high/non-tumor group, 33.1 versus 25.9 months respectively, and this difference was significant (p = 0.024).

Combining tumor and non-tumor and low and high classes, four categories are created. The differences in OS between these categories were significant (Kruskal Wallis p = 0.017; Log Rank = 0.021), with the lowest survival rate in the category low tumor+high non-tumor, i.e., patients with *ERS2*/low expression in tumor tissue and *ERS2*/high expression in non–tumor tissue (median = 16.6 months), and the highest survival rate in high tumor+low non-tumor, i.e., those with *ERS2*/high expression in tumor tissue and *ERS2*/low expression in non–tumor tissue (median = 36.9 months).

None of *ERS2* related variables was included in Cox model because their initial Cox univariate tests exceed 0.2 treshold: p = 0.278, p = 0.990 and p = 0.691 for tumor tissues, non-tumor tissues and ratio tumor/non tumor respectively.

### 3-*CYP19A1*/Aromatase

As reported in **Table 2**, aromatase was measured both at mRNA and protein levels (ng/ml). There were no significant differences between tumor and non-tumor samples when measuring mRNA, p = 0.32. In contrast, comparing protein levels the difference was found to be highly significant, p <0.001. To analyze any relationship between individual mRNA expression levels and protein concentrations a correlation test was performed, but no significant results were obtained (Spearman, p = 0.133).

For survival analysis, again, the total patient sample was divided into low and high (lower and higher than median) expression/concentration groups. Kaplan-Meier tests did not detect any significant differences either for gene expression, tumor samples p = 0.154 (**Figure 1b**), non-tumor samples log-rank p = 0.578, or protein concentration, tumor samples p = 0.266 (**Figure 1b**) and non-tumor samples log-rank p = 0.532.

Although in survival analysis there were no significant differences, **Table 3** shows that the pattern in gene expression and protein concentration was fairly similar: Low expression groups tended to have a longer survival, while high expression groups tended to have shorter survival. Subdividing the total sample into early stage NSCLC (I-II) and late stage (III-IV) NSCLC, there was a significant difference in survival rates within the early-stage cases between low (median = 31.4 months) and high (median = 25.0 months) aromatase gene expression groups: Log Rank = 0.07; Mann-Whitney p = 0.016. There was no such difference when non-tumor tissues samples were compared, or considering aromatase protein concentration.

As happened with *ERS2*, none of *CYP19A1* or aromatase protein related variables was included in final Cox regression model, although one of them passed initial analysis (p<0.200): *CYP19A1* tumor tissues p = 0.966, *CYP19A1* non-tumor tissues p = 0.810, *CYP19A*1 tumor/non tumor ratio p = 0.727, aromatase tumor tissues p = 0.240, aromatase non-tumor tissues p = 0.692 and aromatase tumor/non-tumor ratio p = 0.038.

### 4-Combined gene expression–survival analysis

Recently, it has been shown [22] that combinations of different gene expression results can define in detail the general situation in NSCLC. As previously a given variable is divided into low and high groups, and then, if two variables are combined, four subgroups can be defined; for *ERS1* and *ERS2*, the subgroups are Low-*ERS1*+Low-*ERS2*, Low-*ERS1*+High-*ERS2*, High-*ERS1*+Low-*ERS2* and High *ERS1*+High-*ERS2*. When comparing samples, see **Table 4**, those with the highest expression of the two genes were the ones with highest OS. The difference between Low-*ERS1*+Low-*ERS2* and High *ERS1*+High-*ERS2* groups was significant, Mann-Whitney test p = 0.025, however a Kaplan-Meier OS test was not, p = 0.057 (**Figure 1c**). Medians for subgroups Low-*ERS1*+High-*ERS2* and High-*ERS1*+Low *ERS2* (26.63 and 28.80 months respectively) were close to those for the Low-*ERS1*+Low-*ERS2* group; though the comparisons of the four categories did not detect any significant differences, the pattern obtained tends to suggest that high expression of the two genes is associated with better survival.

**Table 4:** Statistics of overall survival (months) of tumor samples for Low-*ERS1* + Low-*ERS2* group, High-*ERS1* + High-*ERS2* group, Low-*CYP19A1*+Low-aromatase protein and High-*CYP19A1*+High- aromatase protein.

	*ERS1 + ERS2*		*CYP19A1+ ARO*	
	median	N	median	N
Low+Low	27.3	32	30.7	24
High +High	39.4	32	24.1	21
Log Rank	0.057		0.088	
Mann-Whitney	0.025		0.228	

Kaplan-Meier Log Rank and Mann-Whitney p-values when comparing Lower vs. Higher groups are also listed. N: Patient number.

Then, *CYP19A1* expression and aromatase protein concentration were considered. There were no significant differences between Low*CYP19A1*+Low-aromatase protein and High*-CYP19A1*+High aromatase protein groups (**Table 4**). In this case, higher expression and concentrations tended to be associated with shorter survival, although not significant, p = 0.088 (**Figure 1c**).

Finally, expression levels of estrogen receptor genes were combined with aromatase gene expression and protein concentrations, and median OS rates assessed (**Table 5**). Again, four categories were considered in the analysis. The strongest association was found for *ERS1* combined with *CYP19A1* gene expression: the highest OS rates were found for categories High*-ERS1*+Low*-CYP19A1* and High*-ERS1*+High *CYP19A1* (both of them containing the *ERS1-*High expression group), while that for category Low-*ERS1*-high-*CYP19A1* was considerably lower. These differences were significant (Kaplan-Meier Log Rank = 0.0001; Kruskal-Wallis p = 0.011).

**Table 5:** Statistics of overall survival (months) of tumor samples for *ERS1* gene expression, *ERS2* gene expression, *CYP19A1* gene expression and aromatase protein concentration (ARO) for each combination of low and high expression groups (lower and higher than median).

	*ERS1 +CYP19A1*		*ERS1 +ARO*		*ERS2+ CYP19A1*		*ERS2+ARO*	
Class	median	N	median	N	median	N	median	N
Low+Low	31.0	29	26.9	20	30.5	26	28.7	21
High+Low	33.6	19	39.4	23	31.2	22	40.5	25
Low+High	22.2	19	29.7	23	21.4	22	22.3	18
High+High	34.5	29	25.9	19	31.8	26	30.2	21
Log Rank	0.0001		0.383		0.077		0.093	
Kruskal-Wallis	0.011		0.167		0.126		0.049	

Kaplan-Meier Log Rank (Mantel-Cox) and Kruskal-Wallis p-values are listed when comparing these different categories. N: Patient number.

To quantify the extent to which survival was lower in the Low*-ERS1*+High*-CYP19A1* category, the other three categories were merged into one set and a Cox regression was performed. The results were highly significant: p = 0.000007; Exp (B) = 4.041. In this Low-*ER*S1+High-*CYP19A*1 category, a patient's probability of survival was four times lower than for the rest of the patients (regrouped in one set). On the other hand, considering aromatase protein concentration rather than gene expression, the differences were no longer significant.

For the *ERS2*/*CYP19A1* four-category comparison, no significant differences were found (see **Table 5**). The lowest survival corresponded to category Low-*ERS2*+High*-CYP19A1* far from the remaining categories. In fact, repeating the Kaplan-Meier test for Low*-ERS2*+High*-CYP19A1* and other categories individually, the three pairwise comparisons did yield significant differences (Low*-ERS2*+Low*-CYP19A* vs. Low*-ERS2*+High*-CYP19A1* p = 0.031; High*-ERS2*+Low*-CYP19A1* vs. Low*-ERS2*+High*-CYP19A1* p = 0.045; and High*-ERS2*+High*-CYP19A1* vs. Low*-ERS2*+High*-CYP19A1* p = 0.035). As was the case for *ERS1*, the comparison of categories Low*-ERS2*+Low*-CYP19A1*, High*-ERS2*+Low*-CYP19A1* and High*-ERS2*+High*-CYP19A1* regrouped in just one set, with category Low*-ERS2*+High*-CYP19A1*, using Cox regression to quantify survival differences, gave a highly significant result: p = 0.009, Exp (B) = 2.307.

Considering protein concentration in place of *CYP19A1* gene expression, the category with the lowest survival was again Low*-ERS2*+High-aromatase, but rates were dramatically higher for High*-ERS2*+Low-aromatase and notably lower for categories Low*-ERS2*+Low-aromatase and High*-ERS2*+High-aromatase. Differences among the four categories were not significant with the Kaplan-Meier's test (p = 0.093), but they were with Kruskal-Wallis test (p = 0.049).

The *ERS1*+Aromatase and *ERS2*+Aromatase combinations share one interesting feature: High-*ERS1/2*+Low-Aromatase are the subclasses with higher survival in both cases. A Cox regression of High-*ERS1*+Low-Aromatase against the other three *ERS1*+Aromatase combinations was not significant p = 0.114, Exp (B) = 1.89. High-*ERS2*+Low-Aromatase yields the same result p = 0.061 Exp (B) = 2.12.

## Discussion

Although real time RT-PCR has been used previously to determine estrogen receptors expression in lung tumor samples, these studies have been limited due to the small sample size used [23] or because they were restricted to cell cultures [9], [24]. Further, IHC has been the method most commonly employed to detect the presence of ERα and ERβ proteins and variations in their concentration. Research into aromatase has followed a similar pattern [11], [18], [25].

To date, there have been considerable discrepancies in the IHC results reported (even using same antibody, technique and tissue type): detection rates vary from 0 to 80% for ERα, 30 to 100% for ERβ and 60 to 100% for aromatase. Gomez–Fernandez et al. [26], Raso et al. [27] and Miki et al. [28] discussed the problem, establishing a source of these discrepancies: the different antibodies that act against different epitopes. Brueckl et al. [21] and Atmaca et al. [20], published their *ERS1* expression results based on RT-PCR method and human samples (including larger collection of patients), adding a new discrepancy source, the multiplicity of splicing variants of ESR1 mRNAs.

The *ERS1* gene was expressed in 100% of tumor and non-tumor samples. Within the tumor samples there was a significant pattern in survival: those with higher *ERS1*gene expression had longer survival and this contrasts with previous studies. For example, in Fasco et al. [23] no correlation was found, maybe due to the small cohort of patients, while Olivo-Marston et al. [29] reported an inverse correlation between ER-α positive expression and survival in serum samples and in one of the two tissue cohorts studied. The different results may be explained, in part, by the approach to analysis: they took the first tertile as negative expression and the other two as positive. Previous studies have tended to use IHC methods and the results have been very mixed, ranging from no proteins detected to the presence of ER-α associated with poorer survival, alone or in combination with the epidermal growth factor receptor [6], [12], [19], [22], [30], [31]. Other reasons for these differences, besides the aforementioned antibody problem and alternative splicing, are the effects of post-translational regulation on detection [9], [26], [28], [32].

Our *ERS1* expression results related with survival are in agreement with Brueckl et al. [21] and Atmaca et al. [20]. Actually they used an analysis approach similar to what it have been used here, the Delta Cq method, using the median to divide the patients in low and high expression subgroups.

Regarding *ERS2* gene expression, patients with high and low *ERS*2 expression in tumor and non-tumor tissue respectively tend to have higher survival rates; and those with the opposite expression profile (Low *ERS2* in tumor/high *ERS2* in non–tumor tissue) have the poorest prognosis. Some authors did not find any relationship between ER-β protein expression and survival [22], [23], [33] while others, in agreement with our results, observed that presence of the ER-β protein is related to better prognosis. When including in Cox regression multivariate model *ERS2* expression in tumor, non-tumor and its ratio did not yield any significant results. Concerning *CYP19A1* expression, early stage patients expression groups (Low/High) survival results are in concordance with those of Mah et al. [18], although their results were based on IHC methods for protein detection As with *ERS2*, none of *CYP19A1* or aromatase variables were included in Cox multivariate mocel.

Bearing in mind gene/protein expression combinations, High-*ERS1*/High-*ERS2* clearly predicts higher OS and could be used as prognostic factor. Previous reports that used this approach were based on IHC methods and they obtained significant comparisons by combining variables describing the presence of ER-β and aromatase proteins [22], [34], [35].

The most powerful gene combination was found to be Low-*ERS1*+High-*CYP19A1* expressions and this was associated with shorter survival. Having analyzed the four subsets involving *ERS1/CYP19A1*, it seems that Low-*ERS1* is more decisive for survival than High-*CYP19A1* gene expression, since the survival disadvantage is not found when high *CYP19A1* gene expression is combined with high *ERS1* expression, but the OS rate for Low-*ERS1*+/Low-*CYP19A1* expression points to the conclusion that it is the exact combination that results in the shorter OS. The difference in the pattern between expression of the *CYP19A1* and presence of the corresponding protein (**Table 5**) leads us to conclude that something offsets an increase in OS in the category Low-*ERS1*+High*-CYP19A1*, and a decrease in High-*ERS1*/High-*CYP19A1*/High. This may be splicing, post translational modification or another as yet unidentified biological mechanism, though it could also be attributable to the relatively small sample size.

To obtain a comparable predictor of OS based on *ERS2*, the combination Low-*ERS2*+High-*CYP19A1* needed to be set against the three other possible combinations (**Table 5**) grouped in a single set. The hazard ratio from a Cox regression for the patients with Low-*ERS2*+High-*CYP19A1* expression versus the other three categories is 2.307, while the same test for Low-*ERS1*+High-*CYP19A1* expression yielded a hazard ratio of 4.041, underlining the relative strength of *ERS1* levels as a predictor. On the other hand, in the case of *ERS2*/Aromatase protein, the significant differences in survival were noteworthy, a pattern that was not seen with *ERS1*. As with the *ERS1*/CYP19A1 combinations, it may be concluded that Low-*ERS2* is more important than High-*CYP19A1* gene expression when considering the difference in survival between the four subsets. Further, the potential existence of some type of regulation must be considered due to variations in the rates for categories Low-*ERS2*+Low-*CYP19A1*, High-*ERS2*+Low-*CYP19A1* and High-*ERS2*+High-*CYP19A1* and the differences in behavior of *ERS1*and *ERS2* profiles when combined with expression or the product of the *CYP19A1* gene.

In conclusion *ERS1*, alone or in combination with *ERS2* or *CYP19A1*, is the most determining prognostic factor within the analyzed 3 genes taking into account its deregulation in tumor tissues, its relationship with survival, and that the hazard ratio when it is combined with other variables is higher than that for *ERS2*. The origin of the deregulation, which appears to be causing underexpression of *ERS1* RNA has yet to be elucidated. The case of *ERS2* is quite different, since the pattern of patient survival changes considerably when considering expression in non-tumor or tumor tissue. Again, we believe that there is some as yet unknown factor disturbing that process. Further research is required to identify these factors and the level at which they are acting.

## Supporting Information

File S1 Detailed information about ERS1, *ERS2* and CYP19A1 genes transcript length, associated protein length, probe exon-exon boundary, amplicon amplification and protein functionality. **Table S1a**. *CYP19A1* gene transcripts and protein information, adapted from Life Technologies website, for Assay ID Hs00903411_m1. **Table S1b**. *CYP19A1* gene transcripts and protein information, adapted from ENSEMB, for Assay ID Hs00903411_m1. **Table S2a**. *ERS1* gene transcripts and protein information, adapted from Life Technologies website, for Assay ID Hs00174860_m1. **Table S2b**. *ERS1* gene transcripts and protein information, adapted from ENSEMB, for Assay ID Hs00174860_m1. **Table S3a**. *ERS2* gene transcripts and protein information, adapted from Life Technologies website, for Assay ID Hs00230957_m1. **Table S3b**. *ERS2* gene transcripts and protein information, adapted from ENSEMB, for Assay ID Hs00230957_m1.
(DOC)
