# Supplementary material for: Estrogen Receptor 1 Gene Expression and Its Combination with Estrogen Receptor 2 or Aromatase Expression Predicts Survival in Non-Small Cell Lung Cancer
Source: PLoS One. 2014 Oct 13;9(10):e109659. doi: 10.1371/journal.pone.0109659 (PMC4195686; doi:10.1371/journal.pone.0109659)
Supplement: File S1 — Detailed information about ERS1, ERS2 and CYP19A1 genes transcript length, associated protein length, probe exon-exon boundary, amplicon amplification and protein functionality. Table S1a. CYP19A1 gene transcripts and protein information, adapted from Life Technologies website, for Assay ID Hs00903411_m1. Table S1b. CYP19A1 gene transcripts and protein information, adapted from ENSEMB, for Assay ID Hs00903411_m1. Table S2a. ERS1 gene transcripts and protein information, adapted from Life Technologies website, for Assay ID Hs00174860_m1. Table S2b. ERS1 gene transcripts and protein information, adapted from ENSEMB, for Assay ID Hs00174860_m1. Table S3a. ERS2 gene transcripts and protein information, adapted from Life Technologies website, for Assay ID Hs00230957_m1. Table S3b. ERS2 gene transcripts and protein information, adapted from ENSEMB, for Assay ID Hs00230957_m1. (DOC) [file pone.0109659.s001.doc]

**Table S1a**. *CYP19A1* gene transcripts and protein information, adapted from **Life Technologies website**, for Assay ID Hs00903411_m1, produced amplicon 72bp. ExonB= probe exon boundary, Ampli=Amplicon amplification and Funct= functionality.

| Genebank Ref. | length (Bp) | length (AA) | ExonB | Ampli | Funct |
| --- | --- | --- | --- | --- | --- |
| NM_031226.2 | 4531 | 503 | 5/6 | YES | YES |
| NM_000103.3 | 4422 | 503 | 4/5 | YES | YES |
| M18856.1 | 2453 | 419 | 2/3 | YES | Unknown |
| M22246.1 | 2966 | 503 | 3/4 | YES | YES |
| J04127.1 | 2736 | 503 | 3/4 | YES | YES |
| M28420.1 | 2455 | 419 | 2/3 | YES | Unknown |
| X13589.1 | 2966 | 503 | 3/4 | YES | YES |
| Y07508.1 | 2915 | 503 | 3/4 | YES | YES |
| BC056258.1 | 1466 | 359 | 4/5 | YES | Unknown |
| BC020767.1 | 1221 | 359 | 3/4 | YES | Unknown |
| BC022896.1 | 1930 | 181 | 6/7 | YES | NO |
| BC035959.1 | 3014 | 503 | 4/5 | YES | YES |
| BC035714.1 | 1753 | 218 | 3/4 | YES | NO |
| DQ118405.1 | 1653 | 503 | 4/5 | YES | YES |
| BC107785.1 | 2530 | 503 | 4/5 | YES | YES |

Compared to reference sequence NM_000103.3:

M18856.1: Lacks exon 2 first 84 AA.

M28420.1: Lacks exon 2 first 84 AA.

BC056258.1: Lacks 69 AA of exon 9 and complete exon 10.

BC020767.1: Lacks 69 AA of exon 9 and complete exon 10.

BC022896.1: lacks non codifying exon 1, 37 AA of exon 5, exons 6-10.

BC035714.1: Lacks non codifying exon 1, and exon 6-10.

**Table S1b**. *CYP19A1* gene transcripts and protein information, adapted from **ENSEMB,** for Assay ID Hs00903411_m1, produced amplicon 72bp. ExonB= probe exon boundary, Ampli=Amplicon amplification and Func= functionality.

| Name | Transcript ID | Length (bp) | Length (aa) | ExonB | Amp | Func |
| --- | --- | --- | --- | --- | --- | --- |
| CYP19A1-001 | ENST00000396402 | 4417 | 503 | 4/5 | YES | YES |
| CYP19A1-201 | ENST00000260433 | 4409 | 503 | 4/5 | YES | YES |
| CYP19A1-007 | ENST0000039640 | 2388 | 503 | 5/6 | YES | YES |
| CYP19A1-014 | ENST00000559878 | 1958 | 503 | 3/4 | YES | YES |
| CYP19A1-004 | ENST00000405913 | 1697 | 218 | 3/4 | YES | NO |
| CYP19A1-008 | ENST00000453807 | 1062 | 242 | 5/6 | YES | Unknown |
| CYP19A1-015 | ENST00000558328 | 1032 | 283 | 4/5 | YES | Unknown |
| CYP19A1-002 | ENST00000559980 | 993 | 172 | 6/7 | YES | NO |
| CYP19A1-017 | ENST00000561075 | 872 | 244 | 4/- | NO | NO |
| CYP19A1-016 | ENST00000557858 | 832 | 218 | 4/5 | YES | NO |
| CYP19A1-013 | ENST00000405011 | 794 | 166 | 5/6 | YES | Unknown |
| CYP19A1-012 | ENST00000559646 | 592 | 165 | 4/5 | NO | Unknown |
| CYP19A1-020 | ENST00000559653 | 458 | 138 | -/- | NO | NO |
| CYP19A1-011 | ENST00000439712 | 1442 | 352 | 5/6 | Unknown | NO |
| CYP19A1-009 | ENST00000557934 | 1196 | 352 | 4/5 | Unknown | NO |
| CYP19A1-003 | ENST00000492852 | 1758 | --- | NO | NO | --- |
| CYP19A1-018 | ENST00000561066 | 405 | --- | NO | NO | --- |
| CYP19A1-006 | ENST00000478421 | 126 | --- | NO | NO | --- |
| CYP19A1-005 | ENST00000490076 | 766 | --- | NO | NO | --- |
| CYP19A1-019 | ENST00000558066 | 437 | --- | NO | NO | --- |

Compared to CYP19A1-001:

CYP19A1-004: Lacks exons 1, 6, 7 ,8 9,10 and exon 5 fragment. Different first codifying exon.

CYP19A1-008: Extra non codifying exon (exon 2).Lacks 7-10 exons and 17bp of exon 6.

CYP19A1-015: First no codifying exon has a different sequence. Lacks exons 8,9 and 10, and 8 bp in exon 7.

CYP19A1-002: Lacks exons 6-10, shorter exon 5, 3 no codifying exons at 5’ (instead of 1).

CYP19A1-017: Different first no codifying exon. Lacks exon 5, 9, 10 and 112bp of exon 8.

CYP19A1-016: Lacks exons 6-10. Different first no codifying exon. Longer exon 5.

CYP19A1-013: Lacks exons 6-10, shorter exon 5, 2 no codifying exons at 5’ (instead of 1).

CYP19A1-012: Lacks exons 6-10, shorter exon 5, shorter non codifying exon 1.

CYP19A1-020: From the reference sequence only remains exon 4.

CYP19A1-011: Non Stop Decay.

CYP19A1-009: Non Stop Decay.

CYP19A1-003: Non coding exons.

CYP19A1-018: Non coding exons.

CYP19A1-006: Non coding exons.

CYP19A1-005: Non coding exons.

CYP19A1-019: Non coding exons.

**Table S2a**. *ERS1* gene transcripts and protein information, adapted from **Life Technologies website**, for Assay ID Hs00174860_m1. Produced amplicon 62bp. ExonB= probe exon boundary, Ampli=Amplicon amplification and Funct= functionality.

| Genebank Ref. | length (Bp) | length (AA) | ExonB | Ampli | Funct |
| --- | --- | --- | --- | --- | --- |
| NM_000125.3 | 6330 | 595 | 3/4 | YES | YES |
| NM_001122740.1 | 6357 | 595 | 4/5 | YES | YES |
| NM_001122741.1 | 6314 | 595 | 3/4 | YES | YES |
| NM_001122742 | 6466 | 595 | 5/6 | YES | YES |
| M12674.1 | 2092 | 595 | 3/4 | YES | YES |
| X03635.1 | 6450 | 595 | 3/4 | YES | YES |
| U47678.1 | 2966 | 701 | 3/4 | YES | YES |
| BX640939.1 | 5439 | 310 | 3/4 | YES | Unknown |
| BC128573.1 | 2879 | 595 | 3/4 | YES | YES |
| BC128574.1 | 2879 | 595 | 3/4 | YES | YES |
| JF810888.1 | 1788 | 595 | 3/4 | YES | YES |
| AB307713.1 | 1788 | 959 | 3/4 | YES | YES |
| M69296.1 | 2619 | 340 | 3/- | NO | NO |

Compared with reference sequence NM_000125.3:

BX640939.1: Lacks complete codifying exon 1, exon 2 fragment, exon 7 and exon 8.

M69296.1: Lacks exons 4-8.

**Table S2b**. ERS1 gene transcripts and protein information, adapted from **ENSEMB** , for Assay ID Hs00174860_m1. Produced amplicon 62bp. ExonB= probe exon boundary, Ampli=Amplicon amplification and Func= functionality.

| Name | Transcript ID | Length (bp) | Length (aa) | ExonB | Amp | Func |
| --- | --- | --- | --- | --- | --- | --- |
| ESR1-001 | ENST00000206249 | 6455 | 595 | 3/4 | YES | YES |
| ESR1-201 | ENST00000440973 | 6466 | 595 | 5/6 | YES | YES |
| ESR1-202 | ENST00000443427 | 6357 | 595 | 4/5 | YES | YES |
| ESR1-002 | ENST00000338799 | 3335 | 595 | 4/5 | YES | YES |
| ESR1-203 | ENST00000338799 | 5978 | 483 | 4/- | NO | Unknown |
| ESR1-008 | ENST00000427531 | 5437 | 388 | 3/4 | YES | Unknown |
| ESR1-009 | ENST00000406599 | 1251 | 334 | -/- | NO | NO |
| ESR1-204 | ENST00000544394 | 5418 | 310 | 3/4 | YES | Unknown |
| ESR1-007 | ENST00000446550 | 731 | 115 | -/- | NO | NO |
| ESR1-010 | ENST00000415488 | 323 | 107 | -/- | NO | NO |
| ESR1-004 | ENST00000404742 | 624 | 84 | -/- | NO | NO |
| ESR1-003 | ENST00000482101 | 641 | --- | -/- | NO | --- |
| ESR1-005 | ENST00000473497 | 219 | --- | -/- | NO | --- |
| ESR1-006 | ENST00000488573 | 530 | --- | -/- | NO | --- |

Compared to ERS1-001 sequence:

ESR1-203: Lacks codifying exon 4.

ESR1-008: Lacks codifying exons 7 and 8.

ESR1-203: Lacks codifying exons 7 and 8, that have been replaced with other codifying exon.

**Table S3a**. *ERS2* gene transcripts and protein information, adapted from **Life Technologies website,** for Assay ID Hs00230957_m1 produced amplicon 63 bp. ExonB= probe exon boundary, Ampli=Amplicon amplification and Function= functionality.

| Genebank Ref. | length (Bp) | length (AA) | ExonB | Ampli | Function |
| --- | --- | --- | --- | --- | --- |
| NM_001437.2 | 2169 | 530 | 4/5 | YES | YES |
| NM_001040275.1 | 2470 | 495 | 4/5 | YES | YES |
| NM_001040276.1 | 2745 | 495 | 4/5 | Unknown | NO |
| AB006589.1 | 3593 | 495 | 9/10 | YES | YES |
| AB006590.1 | 1740 | 530 | 3/4 | YES | YES |
| X99101.1 | 1560 | 477 | 3/4 | YES | Unknown |
| AF051427.1 | 2011 | 530 | 4/5 | YES | YES |
| AF051428.1 | 2041 | 495 | 4/5 | YES | YES |
| AF060555.1 | 2745 | 513 | 4/5 | YES | Unknown |
| AF074599.1 | 1215 | 381 | 3/4 | YES | Unknown |
| AF124790.1 | 1427 | 323 | 3/4 | YES | Unknown |
| AY438022.1 | 1431 | --- | 2/3 | YES | --- |
| AB209620.1 | 5554 | 503 | 3/4 | YES | Unknown |
| BC024181.2 | 1431 | --- | 4/5 | YES | --- |
| DQ777076.1 | 1634 | 474 | 3/4 | YES | Unknown |
| DQ777077.1 | 1489 | --- | 3/4 | YES | --- |
| DQ838582.1 | 1883 | 481 | 3/4 | YES | Unknown |
| DQ838583.1 | 1596 | 472 | 3/4 | YES | Unknown |

Compared to reference sequence NM_001040275.1:

NM_001040276.1: Nonsense Mediated Decay (NMD).

X99101.1:First exon lacks beginning 53 Aa. Last exon has been replaced with other sequence.

AF060555.1: Last codifying exon has been replaced with other sequence.

AF074599.1: Lacks first exon´s fragment, exon 5 and 6; shares with NM_001437.2 last exon sequence.

AF124790.1: Lacks codifying exons 5-9.

AB209620.1: Lacks last codifying exon. First exon has 32 extras AA at 5’.

BC024181.2: Nonsense Mediated Decay (NMD).

DQ777076.1: Lacks last codifying exon. Instead 5 AA.

DQ838582.1: Lacks last codifying exon. Instead 12 AA.

DQ838583.1: Lacks last codifying exon. Instead 3 AA.

**Table S3b**. *ERS2* gene transcripts and protein information, adapted from **ENSEMBL**, for Assay ID Hs00230957_m1 produced amplicon 63 bp. ExonB= probe exon boundary, Ampli=Amplicon amplification and Func= functionality.

| Name | Transcript ID | Length (bp) | Length (aa) | ExonB | Amp | Func |
| --- | --- | --- | --- | --- | --- | --- |
| ESR2-001 | ENST00000341099 | 2060 | 530 | 4/5 | YES | YES |
| ESR2-204 | ENST00000542956 | 2695 | 513 | 4/5 | YES | YES |
| ESR2-003 | ENST00000554572 | 3588 | 495 | 9/10 | YES | YES |
| ESR2-202 | ENST00000353772 | 2454 | 495 | 4/5 | YES | YES |
| ESR2-002 | ENST00000358599 | 1899 | 495 | 4/5 | YES | YES |
| ESR2-203 | ENST00000358599 | 1914 | 481 | 4/5 | YES | YES |
| ESR2-012 | ENST00000555278 | 1883 | 483 | 3/4 | YES | YES |
| ESR2-011 | ENST00000553796 | 1634 | 474 | 3/4 | YES | YES |
| ESR2-005 | ENST00000557772 | 5458 | 472 | 3/4 | YES | Unknown |
| ESR2-004 | ENST00000556275 | 2695 | 468 | 4/5 | YES | Unknown |
| ESR2-201 | ENST00000267525 | 1788 | 439 | 4/5 | YES | Unknown |
| ESR2-008 | ENST00000344288 | 1427 | 323 | 3/4 | Unknown | NO |
| ESR2-007 | ENST00000555483 | 1436 | --- | 2/3 | YES | --- |
| ESR2-006 | ENST00000554520 | 1215 | --- | 3/4 | YES | --- |
| ESR2-009 | ENST00000555783 | 1196 | --- | 4/5 | UNK | --- |
| ESR2-019 | ENST00000558066 | 306 | --- | -/- | NO | --- |

Compared to ESR2-202:

ESR2-005: Lacks last codifying exon (9).

ESR2-004: Last exon (9) lacks codifying sequence.

ESR2-201: Lacks exons 6(139bp) and 7(134bp).

ESR2-008: Nonsense mediated Decay (NMD).
